# A New Validated HPLC Method for the Simultaneous Determination of 2-phenoxyethanol, Methylparaben, Ethylparaben and Propylparaben in a Pharmaceutical Gel

**DOI:** 10.4103/0250-474X.73906

**Published:** 2010

**Authors:** G. A. Shabir

**Affiliations:** Oxford Brookes University, School of Life Sciences, Headington Campus, Oxford, OX3 0BP, UK

**Keywords:** 2-phenoxyethanol, ethylparaben, HPLC, method validation, methylparaben, pharmaceutical gel, propylparaben

## Abstract

A novel reversed-phase HPLC method has been developed and validated for the simultaneous determination of 2-phenoxyethanol, methylparaben, ethylparaben and propylparaben preservatives. The method uses a Lichrosorb C8 (150×4.6 mm, 5 µm) column and isocratic elution. The mobile phase consisted of a mixture of acetonitrile, tetrahydrofuran and water (21:13:66, v/v/v), pumped at a flow rate of 1 ml/min. The UV detection was set at 258 nm. The method was validated with respect to accuracy, precision (repeatability and intermediate precision), specificity, linearity and range. All the parameters examined met the current recommendations for bioanalytical method validation. The developed method was successfully applied to the determination of commercially available pharmaceutical gel products for these preservatives. The procedure describes here is simple, selective and reliable for routine quality control analysis and stability tests.

Best practices in method development and validation are equally important in the analysis of both active components and preservatives (excipients, inactive components) used in manufacturing of drug products. 2-Phenoxyethanol (C_8_H_10_O_2_, fig. 1a, PhOE), methylparaben (C_8_H_8_O_3_, fig. 1b, MP), ethylparaben (C_9_H_10_O_3_, fig. 1c, EP), and propylparaben (C_10_H_12_O_3_, fig. 1d, PP) are used in single or in combinations in drug, cosmetic and food formulations as antimicrobial preservatives to prevent alteration of product preparations[1].

**Fig. 1:**
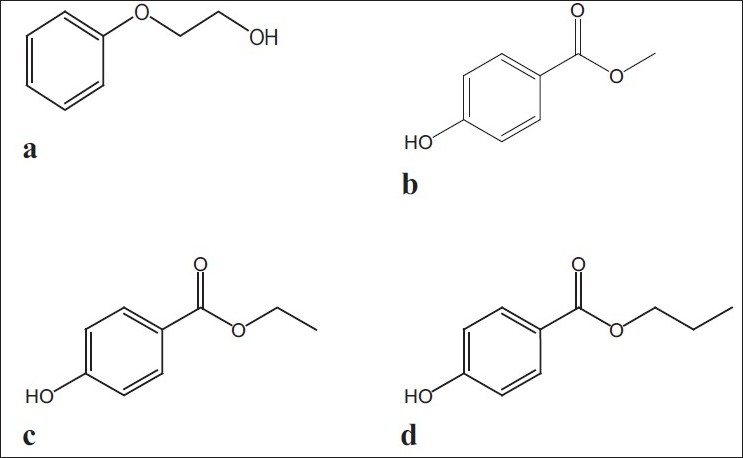
Molecular structures of the separated preservatives The separated preservatives were (a) 2-phenoxyethanol (PhOE), (b) methylparaben (MP), (c) ethylparaben (EP) and d. propylparaben (PP)

Formulators must be fully aware of the procedure for preservative systems in a product need to be analysed to establish their effectiveness throughout shelf life of the product[2]. Many existing analytical procedures are available in literature for the determination of present preservatives studied, either alone or in combination with other drugs by HPLC and other techniques[3–16]. Such a method is important as there seem to be an increasing trend in using combination of preservatives, not only in pharmaceutical formulations but also in food industry and cosmetic products. Moreover, many of the reported methods use complicated and labour-intensive pre-treatment procedures such as steam distillation, multiple-steps and solid phase extractions. Therefore, the purpose of the present study was to develop and validate a new, simple, accurate, and robust reversed-phase HPLC method for the determination of PhOE, MP, EP and PP preservatives in a single chromatographic run suitable for preservatives raw materials, bulk gel samples and finished gel products release. Validating analytical method is a crucial part of successful product development, testing and quality. The determination of preservatives both alone or in formulated products is important and provides a difficult analytical challenge. As a best practice[17–19], in the subsequent investigation, the new and simple reversed-phase HPLC assay method was validated[20] for linearity, precision (repeatability and intermediate precision), accuracy, specificity and robustness. The developed and validated method was applied to the analysis of these preservatives in commercially available pharmaceutical gel products.

## MATERIALS AND METHODS

HPLC-grade acetonitrile and tetrahydrofuran (THF) were obtained from Sigma-Aldrich (Gillingham, UK). 2-Phenoxyethanol (pure> 99%), methylparaben (pure> 99%), ethylparaben (pure> 99%), propylparaben (pure> 99%) and formic acid were also purchased from Sigma-Aldrich (Gillingham, UK). Distilled water was de-ionised by using a Milli-Q system (Millipore, Bedford, MA).

The Knauer HPLC system (Berlin, Germany), consisted of a Knauer pump model 1000, autosampler model 3950, photodiode-array (PDA) detector model 2600 and a vacuum degasser, all controlled by a ClarityChrom software, was used. RP-HPLC analysis was performed isocratically at ambient temperature using a Lichrosorb C8 (150×4.6 mm, 5 µm) column (Jones Chromatography, Hengoed, UK). The mobile phase consisted of a mixture of acetonitrile/tetrahydrofuran/water (21:13:66, v/v/v) adjusted to pH 3.0±0.05 with formic acid was used. The flow rate was 1 ml/min and injection volume was 10 µl. The eluent was monitored with a UV detector set at 258 nm. All samples were diluted with mobile phase.

### Preparation of the standard and sample solutions:

A combined standard stock solution of accurately weighted preservatives PhOE (1.5 g), MP (0.290 g), EP (0.07 g) and PP (0.036 g) was prepared in 100 ml volumetric flask and dissolved in mobile phase (stock). Five millilitre aliquots of PhOE, MP, EP and PP stock solution were added to a 100 ml volumetric flask, and diluted in mobile phase, yielding a final concentration of 750, 145, 35 and 18 µg/ml, respectively. An accurately weighed amount (1.0 g) of gel sample was placed in a 100 ml volumetric flask and dissolved in methanol. Ten millilitre aliquot solution was added to a 100 ml volumetric flask and diluted in 50 ml methanol and volume made up with mobile phase.

### Validation of the method:

The linearity test was performed using five different amounts of PhOE, MP, EP and PP in the range 650-850 µg/ml, 45-245 µg/ml, 20-50 µg/ml and 6-30 µg/ml, respectively. Solutions corresponding to each concentration level were injected in duplicate and linear regression analysis of the PhOE, MP, EP and PP peak area (y) versus PhOE, MP, EP and PP concentration (x) were calculated.

Precision of the method was determined by repeatability (intra-day) and intermediate precision (inter-day variation). Repeatability was examined by analysing six determinations of the same batch of each preservative at 100% of the test concentration. The samples were stored at 30° for 15 days. The RSD of the areas of preservative peak were calculated. Intermediate precision (inter-day variation) was studied by assaying five samples containing the nominal amount of PhOE, MP, EP and PP on different days. Solutions corresponding to each concentration level were injected in duplicate. The RSD values across the system were calculated. Recovery studies may be performed in a variety of ways depending on the composition and properties of the sample matrix. In the present study, three different solutions were prepared with a known added amount of pure PhOE, MP, EP and PP compounds to give a concentration range of 50-150% of that in a test preparation. These solutions were injected in triplicate and percent recoveries of response factor (area/concentration) were calculated. The stability analytical solutions were also evaluated. Sample solutions chromatographed immediately after preparation and then re-assayed after storage at room temperature for 48 h.

## RESULTS AND DISCUSSION

**Fig. 2:**
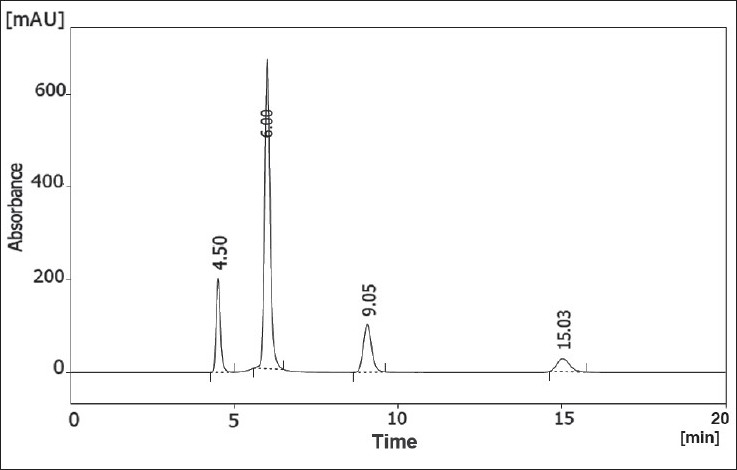
Typical chromatogram of standard mixture of four preservatives PhOE eluted at tR: 4.50 min; MP at tR: 6.00 min; EP at tR: 9.05 min and PP at tR: 15.03 min

The chromatographic separation of PhOE, MP, EP and PP preservatives was carried out in the isocratic mode using a mixture of acetonitrile, tetrahydrofuran and water pH 3.0±0.5 (21:13:66, v/v/v) as mobile phase. The column was equilibrated with the mobile phase flowing at 1 ml/min for about 30 min prior to injection. The column temperature was ambient. Ten microlitres of standards solutions were injected automatically into the column. Subsequently, the liquid chromatographic behaviours of preservatives were monitored with a PDA UV detector at 258 nm. Additionally, preliminary system suitability, precision, linearity, robustness and stability of solutions studies performed during the development of the method showed that the 10 µl injection volume was reproducible and the peak response was significant at the analytical concentration chosen. Chromatograms of the resulting solutions gave excellent separation and resolution (fig. 2).

System suitability test was developed for the routine application of the assay method. Prior to each analysis, the chromatographic system must satisfy suitability test requirements (resolution and repeatability). Peak-to-peak resolution, between each peak measured on a reference solution must be above 1.5. System suitability test was performed from ten replicate injections of a solution containing 750, 145, 35 and 18 µg PhOE, MP, EP and PP/ml, respectively. All peaks were well resolved and the precision of injections for all preservative peaks were acceptable. The percent relative standard deviation (RSD) of the peaks area responses were measured, giving an average between 0.13% and 0.34% (n=10). The tailing factor (T), capacity factor (K) and theoretical plate number (N) were also calculated. The results of system suitability in comparison with the required limits are shown in Table 1. The proposed method met these requirements within the accepted limits[21,22].

**TABLE 1:** SYSTEM SUITABILITY RESULS OF THE PROPOSED ANALYTICAL METHOD

Parameters	Recommended limits	Results			
		PhOE	MP	EP	PP
Retention time (min)	-	4.50	6.00	9.05	15.03
Injection repeatability*	RSD≤1 (%, *n* ≥ 5)	0.34	0.15	0.13	0.26
Resolution (Rs)	R_s_ >1.5	-	10.06	30.70	60.00
Capacity factor (K’)	>2	4.54	5.34	6.45	9.67
Tailing factor (T)	≤2	1.125	1.100	1.133	1.217
Theoretical plate number (N)	>2000	7245	8646	7654	8814

* Ten replicate injections.

For the determination of method robustness within a laboratory a number of chromatographic parameters were evaluated during method development, such as flow rate, column temperature, mobile phase composition and pH, columns from different batches, and the quantitative influence of the variables were determined. For each parameter studied two injections of standard solutions were chromatographed. In all cases the influence of the parameters were found within a previously specified tolerance range. This shows that the method for determination of PhOE, MP, EP and PP was reproducible and robust.

Calibration curves were linear over the concentration range of 650-850 µg/ml for PhOE, 45-245 µg/ml for MP, 20-50 µg/ml for EP and 6-30 µg/ml for PP. The results are presented in Table 2 and show a good correlation between the peak area of analytes and concentration with r > 0.9998. The % RSD were found to be less than 0.22 and 0.52 for intra-day and inter-day precision respectively indicating that the method is reliable and reproducible (Table 3). For determining accuracy, three different solutions were prepared with a known added amount of pure PhOE, MP, EP and PP compounds to give a concentration range of 50-150% of that in a test preparation. These solutions were injected in triplicate. The recovery was 100±2% for all samples with %RSD less than 3% (Table 4).

**TABLE 2:** LINEARITY ASSESSMENT OF THE HPLC METHOD FOR THE ASSAY OF FOUR PRESERVATIVES

Components	Concentration (µg/ml)	Equation for regression line	R^2^
PhOE	650-850*	y = 12432x–7483.2	0.9999
MP	45-245	y = 53084x–179.38	0.9999
EP	20-50	y = 67400x–515	0.9998
PP	6-30	y = 69667x–361.2	0.9998

* K=5; n=2

**TABLE 3:** METHOD VALIDATION RESULTS FOR FOUR PRESERVATIVES

Validation steps	Parameters	Results				Acceptance
		PhOE	MP	EP	PP	criteria
Repeatability	RSD (%, *n* = 6)	0.307	0.097	0.317	0.283	X < 2
Int. precision						
Day 1	RSD (%)	0.329	0.130	0.365	0.401	X < 2
Day 2	RSD (%)	0.242	0.165	0.417	0.372	X < 2
Standard stability (48 h data)	Change in response factor (%)	0.13	0.13	0.13	0.15	X < 2
System suitability	RSD (%, *n* = 6)	0.11	0.15	0.09	0.26	X < 2

RSD: Relative standard deviation.

**TABLE 4:** RECOVERY STUDIES OF THE HPLC METHOD FOR THE ASSAY OF FOUR PRESERVATIVES

	Applied concentration (% of target) (n = 3)			
Components	50	100	150
PhOE	99.86±0.21*	100.00±0.12	99.88±0.14
MP	99.96±0.26	99.97±0.31	98.92±0.18
EP	100.00±0.45	100.00±0.19	99.77±0.36
PP	99.87±0.22	99.78±0.11	99.58±0.28

* The coeffi cient of variation

The LC-PDA isoplot chromatogram was obtained which demonstrated a good separation of the PhOE (RT= 4.50 min), MP (RT= 6.00 min), EP (RT= 9.05 min) and PP (RT= 15.03 min) from each other. A wavelength of 258 nm was found to be the most effective compromise to accomplish the detection and quantification of the four preservative components in a single run. The PhOE, MP, EP and PP peaks are adequately resolved from each other, typical resolution values were >2. Therefore, this method demonstrates acceptable specificity. The stability of analytical solutions was evaluated for 48h. The results given in Table 3 showed there was no significant change (<0.15% response factor) in PhOE, MP, EP and PP concentrations (750, 145, 35 and 18 µg/ml) over this period.

Analytical methods developed for use in quality control laboratories ideally are robust. Retention time for the analytes of interest will not change significantly from day-to-day or from laboratory-to-laboratory if the method is considered robust. To determine the robustness of the chromatographic methodology developed for PhOE, MP, EP and PP, experimental conditions were purposely altered and chromatographic characteristics were evaluated. In particular the pH of the mobile phase was adjusted to 2.5 and 3.5, thus, the normal pH for the method was 3.0. The effected temperature was also studied. Standard solutions were prepared and injected at early 20° and again at 28°. In all cases studied, the retention times of these preservatives (PhOE, MP, EP and PP) were remains same 4.50, 6.00, 9.05 and 15.03 min, respectively. The coefficient of variation for retention time was lass then 1%. Excellent separation was always achieved, indicating that the analytical method remained selective for all components under the measured conditions. A system suitability test was performed to determine the accuracy and precision of the system by injecting six replicate injections of PhOE, MP, EP and PP standard solutions. The RSD of the peak areas responses was measured. The RSD for PhOE (0.11%), MP (0.15%), EP (0.09%) and PP (0.26%) as can be seen in Table 3.

**Fig. 3:**
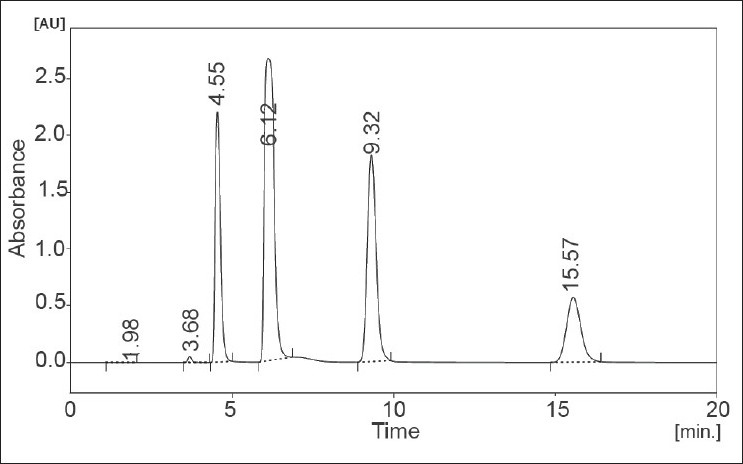
Chromatogram of four preservatives obtained from gel samples PhOE eluted at tR: 4.55 min; MP at tR: 6.12 min; EP at tR: 9.32 min and PP at tR: 15.57 min.

The developed and validated method was applied to the determination of preservatives studied from pharmaceutical gel product. The chromatogram obtained from gel samples is shown in fig. 3. Peak identification of the preservatives in gel sample was based on the comparison between the retention times of standard compounds and was confirmed by spiking known standard compounds to the sample.

A reversed-phase HPLC assay method with UV spectrophotometric detection on a C8 analytical column was developed successfully for the determination of 2-phenoxyethanol, methylparaben, ethylparaben and propylparaben preservatives. The method was validated and the results obtained were accurate and precise with RSD <1% in all cases and no significant interfering peaks were detected. The validated method was successfully applied to the determination of commercially available pharmaceutical gel products for these preservatives. The method can be used for the routine quality control analysis (batch analysis) of compounds in pharmaceutical gel products containing 2-phenoxyethanol, methylparaben, ethylparaben and propylparaben preservatives and the degradation products of the active compounds.
